# Experimental analysis and numerical simulation of bed elevation change in mountain rivers

**DOI:** 10.1186/s40064-016-2714-3

**Published:** 2016-07-13

**Authors:** Truong An Dang, Sang Deog Park

**Affiliations:** 1 Sustainable Management of Natural Resources and Environment Research Group, Faculty of Environment and Labour Safety, Ton Duc Thang University, 19 Nguyen Huu Tho Str., Dist 7, Ho Chi Minh City, Vietnam; 2 Department of Civil Engineering, Gangneung-Wonju National University, Gangneung, Gangwon-Do 210-702 South Korea

**Keywords:** Mountain rivers, Bed load, Morphological changes, Empirical formula, Particle size fractions

## Abstract

Studies of sediment transport problems in mountainous rivers with steep slopes are difficult due to rapid variations in flow regimes, abrupt changes in topography, etc. Sediment transport in mountainous rivers with steep slopes is a complicated subject because bed materials in mountainous rivers are often heterogeneous and contain a wide range of bed material sizes, such as gravel, cobbles, boulders, etc. This paper presents a numerical model that was developed to simulate the river morphology in mountainous rivers where the maximum bed material size is in the range of cobbles. The governing equations were discretized using a finite difference method. In addition, an empirical bed load formula was established to calculate the bed load transport rate. The flow and sediment transport modules were constructed in a decoupled manner. The developed model was tested to simulate the river morphology in an artificial channel and in the Asungjun River section of the mountainous Yangyang Namdae River (South Korea). The simulation results exhibited good agreement with field data.

## Background

Sediment transport in mountain rivers is a complex phenomenon because it is characterized by steep slopes, water depths based on the order of the height, a wide range of bed material sizes, and distinct bed structures. However, our current knowledge of mountain river flow is still improving and progressing due to the lack of understanding of the interrelationship between flow and sediment. In particular, mountainous catchments with the riverbed gradients larger than 0.05 and bed load transport containing a high portion of gravel, cobbles, boulders, and transport capacities during flood events can reach very high values (Chiari 2008; Rickenmann 1990).

In recent decades, numerical models have become useful tools for studying sediment transport problems in mountain rivers. Li and Fullerton (1987) developed a model for simulating channel aggradation and degradation in gravel and cobble-bed rivers. Silvio and Peviani (1989) constructed a numerical model to study the short-and long-term evolution of mountain-rivers. Pianese and Rossi (2005) developed a mathematical model to study the long-term scale changes of a riverbed. In 2004, Papanicolaou developed a new 1D numerical model to calculate flow and sediment transport in steep mountain rivers. According to Mosconi (1988), failure to predict bed loads in mountain river flows may be due to most common equations not considering the morphological peculiarities of study areas in conjunction with their limited capability to cover a wide distribution range of bed material sizes. Bed load equations obtained by several authors on low slopes are rarely applicable to mountain rivers, where the river beds contain wide ranges of bed material sizes, large roughness elements, etc. Therefore, these features largely affect the research results (D’Agostino and Lenzi 1999).

In this paper, a 2D numerical model has been developed to simulate the flow and morphological changes in steep channels where bed materials have large size distributions. The model system consists of a flow module and bed load transport module. The flow module is based on the mass and momentum conservation equations in the Cartesian coordinate system. The sediment transport module only comprises empirical bed load formulas. The river morphology module is based on the sediment continuity equation, and a grain material distribution is applied for individual size fractions. The solution method was implemented in a computer source code and written in structured Fortran 90.

## Numerical method

### Governing equations

By assuming a hydrostatic pressure distribution and neglecting wind shear and Coriolis acceleration, the depth-averaged 2D governing equations are expressed in Cartesian coordinates in the following forms (Ahmadi et al. 2009; Horritt 2004; Lai 2010; Dang and Park 2015).

1 $$\frac{{\partial {\text{h}}}}{{\partial {\text{t}}}} + \frac{\partial }{{\partial {\text{x}}}}({\text{q}}_{\text{x}} ) + \frac{\partial }{{\partial {\text{y}}}}({\text{q}}_{\text{y}} ) = 0$$

2 $$\begin{aligned} \frac{\partial }{{\partial {\text{t}}}}({\text{q}}_{\text{x}} ) + \frac{\partial }{{\partial {\text{x}}}}\left( {\frac{{{\text{q}}_{\text{x}}^{2} }}{\text{h}}} \right) + \frac{\partial }{{\partial {\text{y}}}}\left( {\frac{{{\text{q}}_{\text{x}} {\text{q}}_{\text{y}} }}{\text{h}}} \right)& = - {\text{gh}}\frac{{\partial {\text{Z}}}}{{\partial {\text{x}}}} + \frac{1}{{\uprho {\text{h}}}}\left( {\frac{{\partial {\text{T}}_{\text{xx}} }}{{\partial {\text{x}}}} + \frac{{\partial {\text{T}}_{\text{xy}} }}{{\partial {\text{y}}}}} \right)\\ &\quad - \frac{{\uptau_{\text{bx}} }}{\uprho } + {\text{gh}}\left( {{\text{S}}_{\text{ox}} - {\text{S}}_{\text{fx}} } \right)\end{aligned}$$

3 $$\begin{aligned} \frac{\partial }{{\partial {\text{t}}}}({\text{q}}_{\text{y}} ) + \frac{\partial }{{\partial {\text{x}}}}\left( {\frac{{{\text{q}}_{\text{x}} {\text{q}}_{\text{y}} }}{\text{h}}} \right) + \frac{\partial }{{\partial {\text{y}}}}\left( {\frac{{q_{\text{y}}^{2} }}{\text{h}}} \right) &= - {\text{gh}}\frac{{\partial {\text{Z}}}}{{\partial {\text{y}}}} + \frac{1}{{\uprho {\text{h}}}}\left( {\frac{{\partial {\text{T}}_{\text{yx}} }}{{\partial {\text{x}}}} + \frac{{\partial {\text{T}}_{\text{yy}} }}{{\partial {\text{y}}}}} \right)\\&\quad - \frac{{\uptau_{\text{by}} }}{\uprho }{\text{ + gh}}\left( {{\text{S}}_{\text{oy}} - {\text{S}}_{\text{fy}} } \right) \end{aligned}$$

### Sediment transport equations

In the past, most bed load formulae have been developed and widely used based on laboratory investigations with uniform particle size. Unfortunately, when applied to natural rivers with non-uniform particle size, the calculated bed load transport rates often differ by orders of magnitude and do not exhibit high confidence levels. One of the major causes is often the influence of the local conditions, which are very different from the laboratory conditions where the bed load formulae were constructed. As noted above, mountain rivers is a dominant and fundamental process in the hydrodynamic rivers, and often have non-uniform particle size distributions, the particle sizes are divided into several fractions.

**Fig. 1:**
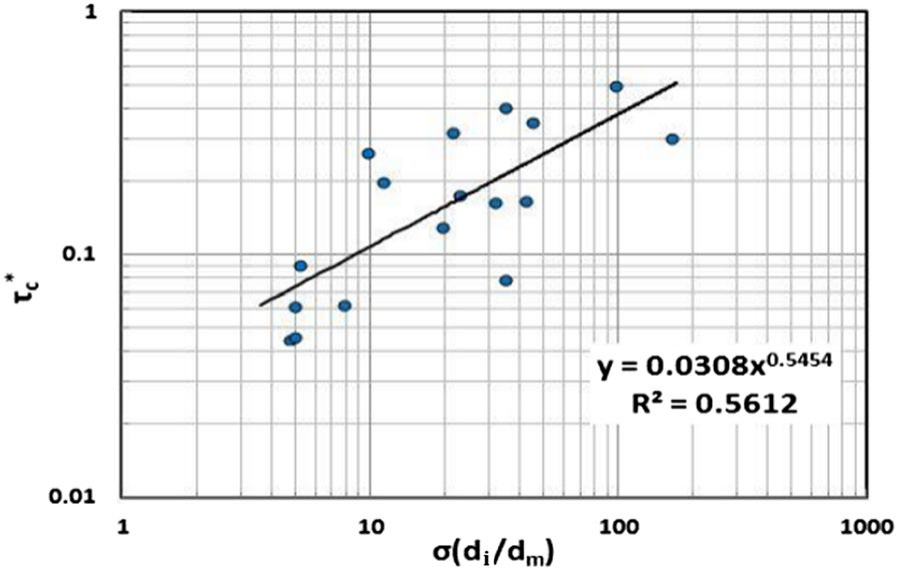
Relationship between $\uptau_{\text{c}}^{*}$ and σ (d_i_/d_m_) (Park et al. 2013)

Therefore, in this study, only bed load is used and no suspended sediment is included. An empirical formula has been developed by the Department of Civil Engineering, Gangneung-Wonju National University (Park et al. 2013), based on investigation data from mountain rivers in South Korea. The bed load transport formula can be applied to mountain rivers in which the channel bed materials are non-uniform and includes, gravel, and cobble. The bed load transport formula is given as follows:

4 $${\text{q}}_{\text{sb}}^{*} = \sum\limits_{{{\text{i}} = 1}}^{\text{N}} {{\text{q}}_{\text{sbi}} }$$

5 $${\text{q}}_{\text{sb}}^{*} = \frac{{{\text{q}}_{\text{sb}} }}{{\upgamma \sqrt {{\text{g}}\left( {\upsigma_{\text{s}} - 1} \right){\text{d}}^{3} } }}$$

6 $${\text{q}}_{\text{sb}}^{*} = 0.00157\uptau_{\text{i}}^{*0.418} \left( {\uptau_{\text{i}}^{*} - \uptau_{\text{ci}}^{*} } \right)^{0.307}$$

7 $$\uptau_{\text{ic}}^{*} = 0.0308\left( {\uptau_{\text{b}} \frac{{{\text{d}}_{\text{i}} }}{{{\text{d}}_{\text{m}} }}} \right)^{0.545}$$

8 $$\uptau_{\text{i}}^{*} = \frac{{\uptau_{\text{b}} }}{{\upgamma \left( {\upsigma_{\text{s}} - 1} \right){\text{d}}_{\text{i}} }}$$

where ${\text{q}}_{\text{sb}}^{*}$ is the bed load discharge, ${\text{q}}_{\text{sb}}^{*}$ is the bed load transport capacity, σ_s_ is the specific gravity, g is the gravitational acceleration, d is the particle size of the bed material, $\uptau_{\text{ci}}^{*}$ is the dimensionless critical Shields stress of incipient motion, and τ_b_ is the bed shear stress. τ_b_, σ_b_, and σ_s_ are expressed as follows:

9 $$\begin{aligned} \uptau_{\text{b}} & = \upgamma {\text{HS}}; \\ \upsigma_{\text{b}} & = \left( {\frac{{{\text{d}}_{84} }}{{{\text{d}}_{16} }}} \right)^{1/2} ; \\ \upsigma_{\text{s}} & = \frac{{\upgamma_{\text{s}} }}{\upgamma } \\ \end{aligned}$$

where H is the water depth, d is the particle size of the bed material, S is the stream slope, γ_s_ is the specific weight of sediment, and γ is the specific weight of water (Fig. 1).

### Bed level variation

By considering only the bed load transport, the 2D sediment continuity equation may be written as follows:

10 $$\frac{{\partial {\text{Z}}_{\text{b}} }}{{\partial {\text{t}}}} + \frac{1}{{1 - {\text{p}}}}\left( {\frac{{\partial {\text{q}}_{\text{bx}} }}{{\partial {\text{x}}}} + \frac{{\partial {\text{q}}_{\text{by}} }}{{\partial {\text{y}}}}} \right) = 0$$

where Z_b_ is the local bed elevation, p is the porosity of the bed material, ∂Z_b_ is the change in the local bed level during the time interval ∂t, and q_bx_ and q_by_ are the x-and y-components of the bed load transport per unit width, respectively.

The components of the bed load transport in the x- and y-directions are related to the bed load q_b_ as follows:

11 $${\text{q}}_{\text{bx}} = {\text{q}}_{\text{sb}} \cos \upalpha$$

12 $${\text{q}}_{\text{by}} = {\text{q}}_{\text{sb}} \sin \upalpha$$

where q_sb_ appears in Eqs. (11) and (12) and is calculated by Eq. (5). In Eqs. (11) and (12), α is calculated by Eq. (13) and corresponds to the directional angle of bed load transport in the x- and y-planes, as follows:

13 $$\tan \upalpha = \frac{{\sin \upbeta - \frac{1}{{{\text{f}}_{s} \uptau^{*} }}\frac{{\partial {\text{z}}_{\text{b}} }}{{\partial {\text{x}}}}}}{{\cos \upbeta - \frac{1}{{{\text{f}}_{\text{s}} \uptau^{*} }}\frac{{\partial {\text{z}}_{\text{b}} }}{{\partial {\text{y}}}}}}$$

where f_s_ is the sediment shape factor.

Several studies have been carried out to propose a formulation for f_s_, such as Ikeda (1982), Kovacs and Parker (1994), and Zimmerman and Kennedy (1978). Talmon (1992) used the following expression of the sediment shape factor:

14 $${\text{f}}_{\text{s}} = 9\left( {\frac{{{\text{d}}_{50} }}{\text{h}}} \right)^{0.3} \sqrt {\uptau^{*} }$$

where d_50_/h is the relative roughness parameter. Note that this formulation controls the effect of gravity on the sediment particles. In Eq. (13), the term β is calculated as follows.

15 $$\upbeta = \tan^{ - 1} \left( {\frac{\text{v}}{\text{u}}} \right) - \tan^{ - 1} \left( {\frac{\text{A}}{{{\text{r}}_{\text{s}} }}{\text{h}}} \right)$$

The terms A and r_s_ in Eq. (15) are given as follows.

16 $${\text{A}} = \frac{2}{{{\text{k}}^{2} }}\left[ {1 - \frac{{{\text{n}}\sqrt {\text{g}} }}{{{\text{kh}}^{1/6} }}} \right]$$

17 $${\text{r}}_{\text{s}} = \frac{{\left| {{\text{u}}_{\text{i}} } \right|^{3} }}{{\left[ {{\text{u}}^{2} \frac{{\partial {\text{v}}}}{{\partial {\text{x}}}} + {\text{uv}}\left( {\frac{{\partial {\text{v}}}}{{\partial {\text{y}}}} - \frac{{\partial {\text{v}}}}{{\partial {\text{x}}}}} \right) - {\text{v}}^{2} \frac{{\partial {\text{u}}}}{{\partial {\text{y}}}}} \right]}}$$

Rozovskii (1957) and Engelund (1974) suggested that the value of A was 11 and the value of r_s_ was seven.

The transverse bed slope was small and had little effect on the flow calculations. Therefore, in the research, the effect of transverse bed slope is ignored.

## Discretization of governing equations

**Fig. 2:**
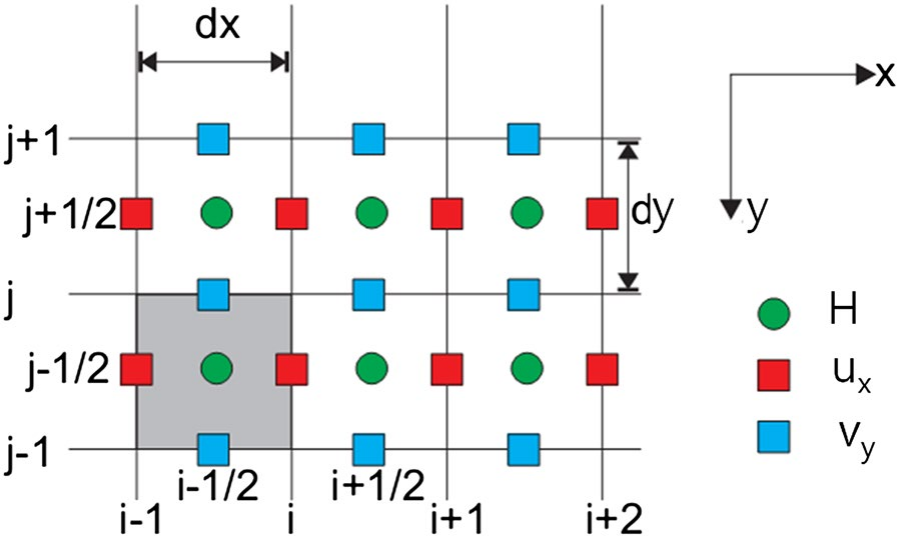
Staggered grid with boundary cells

The governing equations are discretized in the computational domain using an finite difference method (FDM) and a staggered grid in Cartesian coordinates. In this explicit difference formulation, a first-order approximation was used for the temporal derivative (∆t). Second-order central difference approximations were used for space discretization (∆x, ∆y), where the water depth (h) is defined at the primary grid center (i, j) and the velocity components (u, v) are defined at the cell faces of the secondary grid (i + 1/2, j + 1/2) (Fig. 2) (Hung et al. 2009; Jia and Wang 2001; Dang and Park 2015).

Equation (1) is applied at grid point (i, j), yielding the following finite difference equation.

18 $${\text{H}}_{{{\text{i}},{\text{j}}}}^{{{\text{T}} + 1}} = {\text{H}}_{{{\text{i}},{\text{j}}}}^{\text{T}} - \frac{{\Delta {\text{t}}}}{{\Delta {\text{x}}}}\left[ {\left( {{\text{q}}_{\text{x}} } \right)_{{{\text{i}} + \tfrac{1}{2},{\text{j}}}}^{{{\text{T}} + 1}} - \left( {{\text{q}}_{\text{x}} } \right)_{{{\text{i}} - \tfrac{1}{2},{\text{j}}}}^{{{\text{T}} + 1}} } \right] - \frac{{\Delta {\text{t}}}}{{\Delta {\text{y}}}}\left[ {\left( {{\text{q}}_{\text{y}} } \right)_{{{\text{i}},{\text{j}} + \tfrac{1}{2}}}^{{{\text{T}} + 1}} - \left( {{\text{q}}_{\text{y}} } \right)_{{{\text{i}},{\text{j}} - \tfrac{1}{2}}}^{{{\text{T}} + 1}} } \right]$$

The x-momentum equation is applied to the secondary grid at grid point (i + 1/2, j).

19 $$\begin{aligned} {\text{P}}_{{{\text{I}} + \frac{1}{2},{\text{J}}}}^{{{\text{T}} + 1}} & = {\text{P}}_{{{\text{I}} + \frac{1}{2},{\text{J}}}}^{\text{T}} - \frac{{\Delta {\text{t}}}}{{\Delta {\text{x}}}}{\text{H}}_{{{\text{I}} + \frac{1}{2},{\text{J}}}} \left[ {H_{{{\text{I}} + 1,{\text{J}}}}^{\text{T}} - {\text{H}}_{{{\text{I}},{\text{J}}}}^{\text{T}} } \right] + \frac{{\Delta {\text{t}}}}{{\Delta {\text{x}}}}\frac{1}{{{\text{H}}_{{{\text{I}} + \frac{1}{2},{\text{J}}}} }}\left[ {\left( {{\text{P}}_{{{\text{I}} + 1,{\text{J}}}}^{\text{T}} } \right)^{2} - \left( {{\text{P}}_{{{\text{I}},{\text{J}}}}^{\text{T}} } \right)^{2} } \right] \\& \quad - {\text{g}}\frac{{\Delta {\text{t}}}}{{\Delta {\text{y}}}}\frac{1}{{{\text{H}}_{{{\text{I}} + \frac{1}{2},{\text{J}}}} }}\left[ {\left( {\text{PQ}} \right)_{{{\text{I}} + \frac{1}{2},{\text{J}} + \frac{1}{2}}}^{\text{T}} - \left( {\text{PQ}} \right)_{{{\text{I}} + \frac{1}{2},{\text{J}} - \frac{1}{2}}}^{\text{T}} } \right] \\ & \quad - {\text{g}}\frac{{\Delta {\text{t}}}}{{\Delta {\text{x}}}}{\text{H}}_{{{\text{I}} + \frac{1}{2},{\text{J}}}} \left[ {{\text{Z}}_{{{\text{I}} + 1,{\text{J}}}}^{\text{T}} - {\text{Z}}_{{{\text{I}},{\text{J}}}}^{\text{T}} } \right] - \Delta {\text{tgn}}^{2} \left( {H_{{{\text{I}} + \frac{1}{2},{\text{J}}}}^{\text{T}} } \right)^{{ - \frac{7}{3}}} \\&\quad \;\; P_{{{\text{I}} + \frac{1}{2},{\text{J}}}}^{\text{T}} \sqrt {({\text{P}}^{2} )_{{{\text{I}} + \frac{1}{2},{\text{J}}}}^{\text{T}} + ({\text{Q}}^{2} )_{{{\text{I}},{\text{J}} + \frac{1}{2}}}^{\text{T}} } \\ \end{aligned}$$

Finally, the y-component is applied at grid point (i, j + 1/2), yielding the following finite difference equation.

20 $$\begin{aligned} {\text{Q}}_{{{\text{I}},{\text{J}} + \frac{1}{2}}}^{{{\text{T}} + 1}} & = {\text{Q}}_{{{\text{I}},{\text{J}} + \frac{1}{2}}}^{\text{T}} - \frac{{\Delta {\text{t}}}}{{\Delta {\text{x}}}}\frac{1}{{{\text{h}}_{{{\text{I}},{\text{J}} + \frac{1}{2}}}^{\text{T}} }}\left[ {\left( {\text{PQ}} \right)_{{{\text{I}} + \frac{1}{2},{\text{J}} + \frac{1}{2}}}^{\text{T}} - \left( {\text{PQ}} \right)_{{{\text{I}} - \frac{1}{2},{\text{J}} + \frac{1}{2}}}^{\text{T}} } \right] - \frac{{\Delta {\text{t}}}}{{\Delta {\text{y}}}}\frac{1}{{{\text{H}}_{{{\text{I}},{\text{J}} + \frac{1}{2}}}^{\text{T}} }}\left[ {\left( {{\text{Q}}_{{{\text{I}},{\text{J}} + 1}}^{\text{T}} } \right)^{2} - \left( {{\text{Q}}_{{{\text{I}},{\text{J}}}}^{\text{T}} } \right)^{2} } \right] \\ & \quad - {\text{g}}\frac{{\Delta {\text{t}}}}{{\Delta {\text{y}}}}{\text{H}}_{{{\text{I}},{\text{J}} + \frac{1}{2}}}^{\text{T}} \left[ {{\text{H}}_{{{\text{I}},{\text{J}} + 1}}^{\text{T}} - {\text{H}}_{{{\text{I}},{\text{J}}}}^{\text{T}} } \right] - {\text{g}}\frac{{\Delta {\text{t}}}}{{\Delta {\text{y}}}}{\text{H}}_{{{\text{I}},{\text{J}} + \frac{1}{2}}}^{\text{T}} \left[ {{\text{Z}}_{{{\text{I}},{\text{J}} + 1}}^{\text{T}} - {\text{Z}}_{{{\text{I}},{\text{J}}}}^{\text{T}} } \right] - {\text{gn}}^{2} \Delta {\text{t}}\left( {{\text{H}}_{{{\text{I}},{\text{J}} + \frac{1}{2}}}^{\text{T}} } \right)^{{ - \frac{7}{3}}} {\text{Q}}_{{{\text{I}},{\text{J}} + \frac{1}{2}}}^{\text{T}} \sqrt {({\text{P}}^{2} )_{{{\text{I}} + \frac{1}{2},{\text{J}}}}^{\text{T}} + ({\text{Q}}^{2} )_{{{\text{I}},{\text{J}} + \frac{1}{2}}}^{\text{T}} } \\ \end{aligned}$$

For this purpose, the following approximations are used.

$$\left( {{\text{u}}^{2} } \right)_{{{\text{I}} + 1,{\text{J}}}} = \frac{1}{4}\left( {{\text{u}}_{{{\text{I}} + \frac{3}{2},{\text{J}}}} + {\text{u}}_{{{\text{I}} + \frac{1}{2},{\text{J}}}} } \right)^{2} ;\quad \left( {{\text{u}}^{2} } \right)_{{{\text{I}},{\text{J}}}} = \frac{1}{4}\left( {{\text{u}}_{{{\text{I}} + \frac{1}{2},{\text{J}}}} + {\text{u}}_{{{\text{I}} - \frac{1}{2},{\text{J}}}} } \right)^{2}$$

$$\left( {{\text{v}}^{2} } \right)_{{{\text{I}},{\text{J}} + 1}} = \frac{1}{4}\left( {{\text{v}}_{{{\text{I}},{\text{J}} + \frac{3}{2}}} + {\text{v}}_{{{\text{I}},{\text{J}} + \frac{1}{2}}} } \right)^{2} ;\quad \left( {{\text{v}}^{2} } \right)_{{{\text{I}},{\text{J}}}} = \frac{1}{4}\left( {{\text{v}}_{{{\text{I}},{\text{J}} + \frac{1}{2}}} + {\text{v}}_{{{\text{I}},{\text{J}} - \frac{1}{2}}} } \right)^{2}$$

$$\left( {\text{uv}} \right)_{{{\text{I}} + \frac{1}{2},{\text{J}} + \frac{1}{2}}} = \frac{1}{4}\left( {{\text{u}}_{{{\text{I}} + \frac{1}{2},{\text{J}}}} + {\text{u}}_{{{\text{I}} + \frac{1}{2},{\text{J}} + 1}} } \right)\left( {{\text{v}}_{{{\text{I}},{\text{J}} + \frac{1}{2}}} + {\text{u}}_{{{\text{I}} + 1,{\text{J}} + \frac{1}{2}}} } \right)$$

$$\left( {\text{uv}} \right)_{{{\text{I}} + \frac{1}{2},{\text{J}} - \frac{1}{2}}} = \frac{1}{4}\left( {{\text{u}}_{{{\text{I}} + \frac{1}{2},{\text{J}}}} + {\text{u}}_{{{\text{I}} + \frac{1}{2},{\text{J}} - 1}} } \right)\left( {{\text{v}}_{{{\text{I}},{\text{J}} - \frac{1}{2}}} + {\text{u}}_{{{\text{I}} + 1,{\text{J}} - \frac{1}{2}}} } \right)$$

$$\left( {\text{uv}} \right)_{{{\text{I}} - \frac{1}{2},{\text{J}} + \frac{1}{2}}} = \frac{1}{4}\left( {{\text{u}}_{{{\text{I}} - \frac{1}{2},{\text{J}}}} + {\text{u}}_{{{\text{I}} - \frac{1}{2},{\text{J}} + 1}} } \right)\left( {{\text{v}}_{{{\text{I}},{\text{J}} + \frac{1}{2}}} + {\text{u}}_{{{\text{I}} - 1,{\text{J}} + \frac{1}{2}}} } \right)$$

The sediment continuity equation given by (10) is discretized as follows.

21 $$\frac{{{\text{Z}}_{{{\text{bi}},{\text{j}}}}^{{{\text{T}} + 1}} - {\text{Z}}_{{{\text{bi}},{\text{j}}}}^{\text{T}} }}{{\Delta {\text{t}}}} = - \frac{1}{1 - \lambda }\left[ {\frac{{{\text{q}}_{{{\text{bi}} + \tfrac{1}{2},{\text{j}}}}^{{{\text{T}} + 1}} - {\text{q}}_{{{\text{bi}} - \tfrac{1}{2},{\text{j}}}}^{{{\text{T}} + 1}} }}{{\Delta {\text{x}}}}} \right] - \frac{1}{1 - \lambda }\left[ {\frac{{{\text{q}}_{{{\text{bi}},{\text{j}} + \tfrac{1}{2}}}^{{{\text{T}} + 1}} - {\text{q}}_{{{\text{bi}},{\text{j}} - \tfrac{1}{2}}}^{{{\text{T}} + 1}} }}{{\Delta {\text{y}}}}} \right]$$

In Eq. (18), the discharge components are specified at time step (T + 1). The momentum equations [Eqs. (19) and (20)] in the x-and y-directions are solved first to provide P_I+1/2,J_ and Q_I,J+1/2_, (with P = u·h and Q = v·h) the values of the discharge components at time step (T + 1). The water depth at the time step (T + 1) in Eq. (18) is then solved. Equation (18) is solved iteratively to determine the value of H_i,j_ at time step (T + 1) over the entire domain. Subsequently, Eqs. (19) and (20) are used to compute the velocity components at time step (T + 1) over the entire domain (Horritt 2004; Hung et al. 2009; Matyka 2004; Dang and Park 2015).

**Fig. 3:**
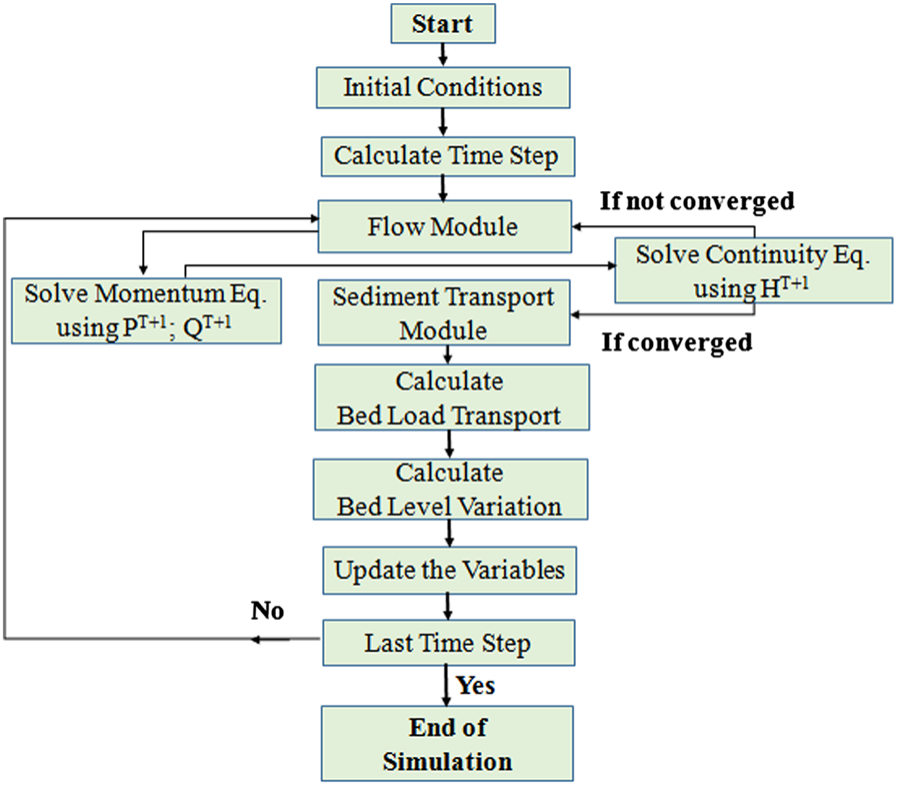
Flow chart of the numerical simulation model

The steps in the process of solving the flow and bed load equations are listed below and are illustrated in Fig. 3.

Step 1: Initializing all the variables. This step usually corresponds to time T_0_. In this step, the values of the water depth and flow field within the computational domain and at the boundaries are specifically established. It is assumed that the velocity components and water depth are known at time T_0_ and that the boundary conditions of the velocity components and water depth are given.
Step 2: Partial differential Eqs. (18), (19), and (20) for the flow and Eq. (21) for sediment continuity are solved with the finite difference code. Discretized equations are obtained for the shallow water and sediment continuity equations using the staggered numerical grid. The initial and boundary conditions used to solve the momentum Eqs. (19) and (20) and the continuity Eq. (18), i.e., the values of u, v, and h at time T + 1, are determined at every interior node (I = 2,…, N). The values of the dependent variables u and v at the boundary nodes 1 and N + 1 are determined using the boundary conditions. The values of the dependent variables that are not specified through boundary conditions can be determined by extrapolation of the interior points or equivalently by approximation of the derivatives at fictitious boundary points. We then obtain the corrected water depth and (u, v) velocity components at every interior node in the computational domain.
Step 3: The velocity components are calculated at time step T = T_0_ + ΔT until a converged solution is obtained. In this step, convergence criteria must be checked because the scheme used in this research is an iterative scheme. Then, the velocity components and water depth are updated with their corresponding values.
Step 4: The water depth and velocity components are used to calculate the dimensionless particle diameter, dimensionless Shields stress, dimensionless critical Shields stress, critical shear stress, boundary shear stress, etc. Finally, the dimensionless particle diameter is calculated.
Step 5: The parameters calculated in Step 4 are used to calculate the bed load transport rate.
Step 6: Erosion and deposition are calculated using the sediment continuity Eq. (21) to determine the bed level variation and update the new water depth if the channel bed has changed.
Step 7: Return to Step 2 and repeat the preceding calculation until the specified final time. If a steady state solution is required, a specified convergence criterion must be satisfied.
Step 8: The last step in the calculation process involves storing and updating variables at each time step, moving to the next time step, and repeating Step 2 through Step 7.

### Stability conditions

In explicit difference schemes such as the MacCormack, Lax-Wendroff, and Marker and Cell schemes, the magnitude of the time step is governed by the CFL stability condition (Bellos and Hrissanthou 2003; Chow and Ben-Zvi 1973; McKee et al. 2004, 2008; Paulo et al. 2007; Rao 2003). In this study, the following expression for the CFL stability condition was used:

22 $$\Delta {\text{t}} = \upalpha \frac{{\Delta {\text{x}}^{2} \Delta {\text{y}}^{2} }}{{\left( {\sqrt {{\text{u}}^{2} + {\text{v}}^{2} } + \sqrt {\text{gh}} } \right)\left( {\sqrt {\Delta {\text{x}}^{2} + \Delta {\text{y}}^{2} } } \right)}}$$

where ∆t is the time increment; ∆x and ∆y are the grid spacings; u and v are the velocity components in the x- and y-directions, respectively; h is the water depth; g is the acceleration of gravity; and α is the coefficient (α ≤ 1).

## Application of model verification and discussion

To investigate the applicability of the developed model, the present model has been tested in two experimental cases. The first case was obtained from the Large Scale Hydraulic Models of the University of Calabria, Italy (Bellos and Hrissanthou 2003; Bor 2008; Miglio et al. 2009). The second was obtained from a flood event in the Asungjun River.

Numerical models can be calibrated by comparing measured and computed results and adjusting the empirical coefficients in the associated empirical relationships. By a trial and error procedure, the agreement between calculations and measurements can be satisfied. However, this procedure is difficult to apply because of the lack of input data, especially for simulating flow and sediment transport in natural rivers. Several researchers have determined the goodness of fit of hydrodynamic models by computing the root mean square differences (RMSD) and mean absolute errors (MAE) between observed and simulated results.

In this study, the expression for calculating the RMSD (see Eq. 23) was selected to determine the error between the calculated results and the measured data, minimizing the RMSD error between the calculated results and measured data. The RMSD error is defined as follows.

23 $${\text{RMSD}} = \sqrt {\frac{1}{\text{n}}\sum\limits_{1}^{\text{N}} {\left( {{\text{Simulated}} - {\text{Measured}}} \right)^{2} } }$$

The root mean square deviation provides a measure of variance between the observed and simulated results.

The expression used to calculate MAE is defined as follows:

24 $${\text{MAE}} = \frac{100}{\text{N}}\sum {\left| {\frac{{{\text{U}}_{\text{M}} - {\text{U}}_{\text{C}} }}{{{\text{U}}_{\text{M}} }}} \right|}$$

where U_M_ is the measured value, U_C_ is the calculated value, and N is the total number of samples.

### Experimental data from a seal aggradation test

#### Model setup

**Fig. 4:**
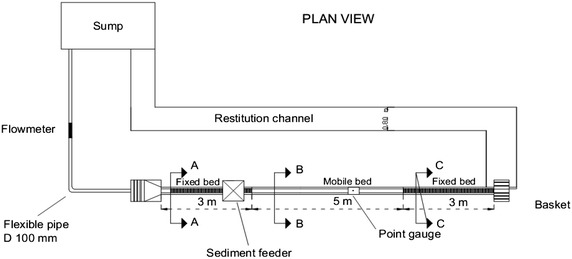
Illustration of the experimental flume at the University of Calabria (Lai 2010)

First, the developed model was tested by simulating the artificial channel. An experimental facility was installed at the Large-Scale Hydraulic Model at the University of Calabria, Italy (Fig. 4). The experiment was conducted and established with the following conditions:

- Width of the artificial channel: 0.194 m;
- Length of the artificial channel: 5.0 m;
- Water depth: 4.3 cm;
- Flow discharge: 0.0242 m^3^/s;
- Bed slope: 1.0 %;
- Grid spacing: Dx = Dy = 0.01 m;
- Median diameter: D_50_ = 3.0 mm;
- Porosity: p = 0.35;
- Manning’s coefficient: 0.015.

The experimental flume data were measured over a period of 30 min with a time step of 1.0 s.

#### Results and discussion of the seal aggradation test case

**Fig. 5:**
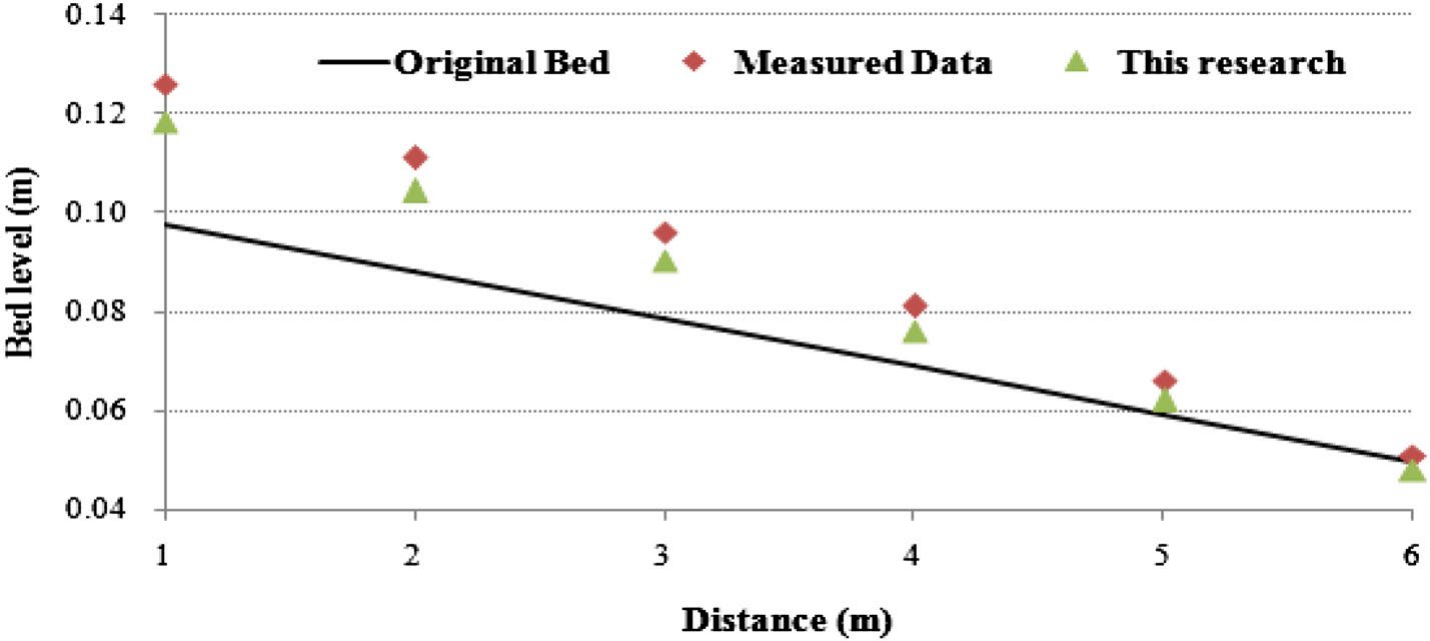
Variations in longitudinal bed profiles with time at T = 30 min

The comparison of the bed level variation showed small differences between the measured and predicted results. The simulation result was in agreement with the measured data during the simulation period (Fig. 5). In general, a good correspondence was observed between the aggradation and degradation tendencies along the experimental flume, with an RMSD of 0.53. Similarly, the validation of the calculated and measured bed level variations using the Nash–Sutcliffe criterion (Nash and Sutcliffe 1970) corresponds to a value of 0.98. This confirms that the simulation model of bed level variation is quite accurate.

### Model test of the Asungjun River section

#### Model setup

**Fig. 6:**
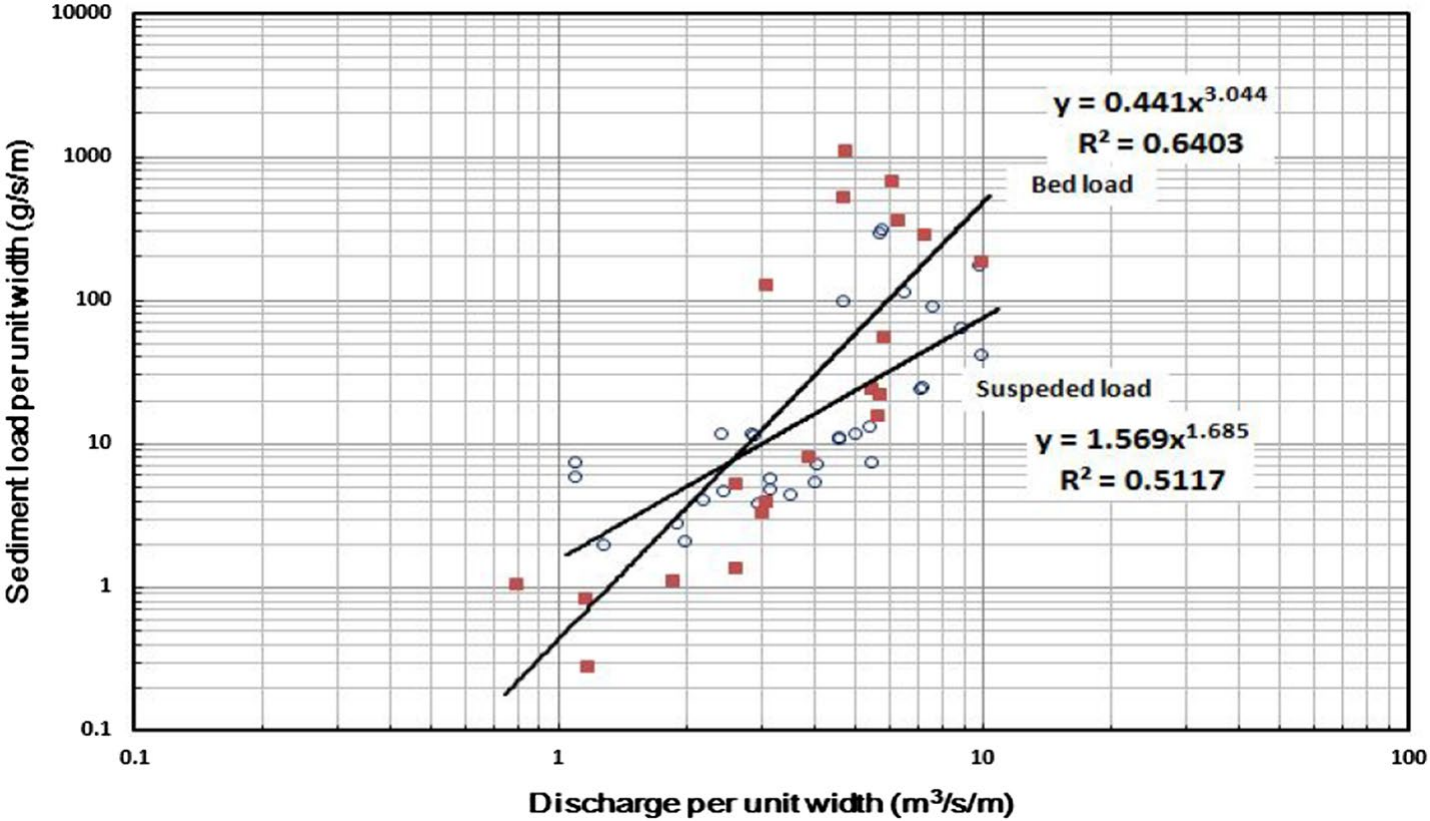
Discharge of bed load and suspended sediment in the study area

Next, the developed model was tested by simulating flood events in the Asungjun River section. Bed loads in mountainous rivers play important roles in the evolution of river beds. During the flood season in the studied river section, the bed material is predominantly sand, gravel, and cobble. The main mode of sediment transport when the unit discharge is more than 2.5 m^3^/s is bed load transport (Fig. 6).

The simulation was established with the following conditions:

- Width of the artificial channel: 250 m;
- Length of the artificial channel: 600 m;
- Grid spacing: Dx = Dy = 1.0 m;
- Porosity: p = 0.40.

**Fig. 7:**
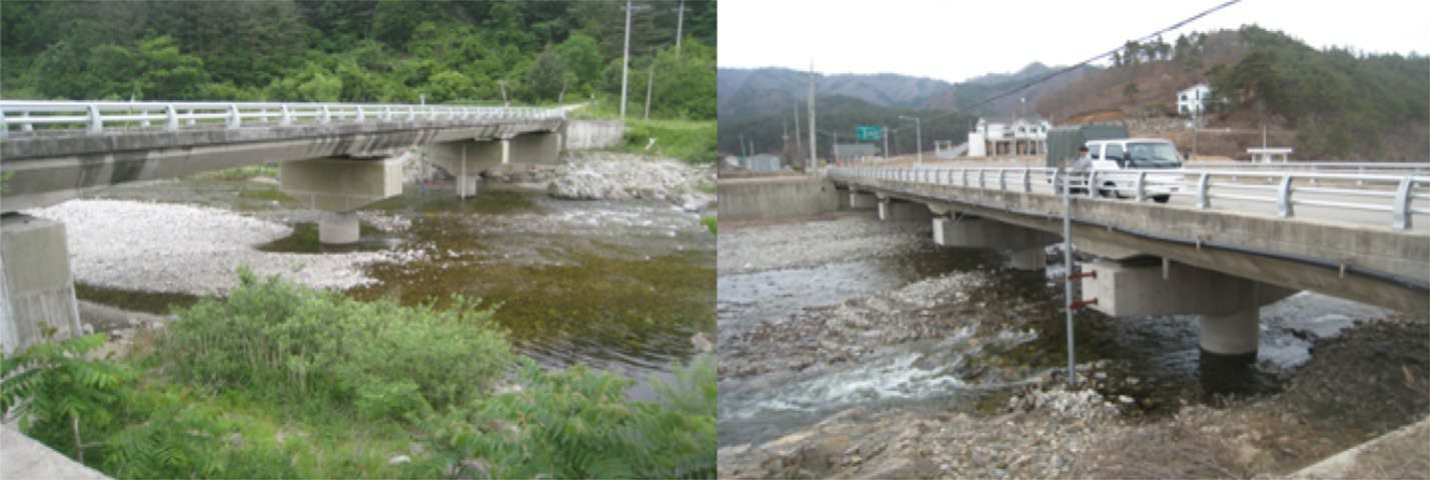
Locations of water level stations at the inflow (*left*) and outflow (*right*) boundaries

Hydrographs of the water level at the upstream boundary (Fig. 7) were provided because the flow in steep mountain rivers is often critical; however, no hydrographs were provided for the water level at the downstream boundary.

**Fig. 8:**
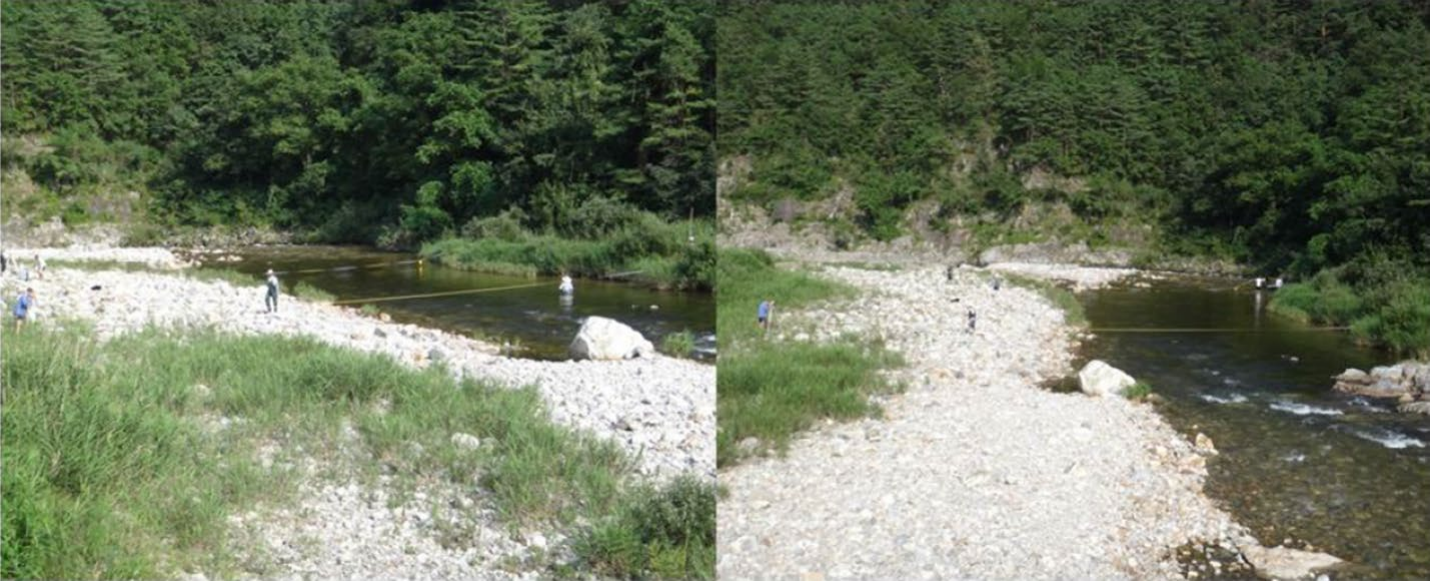
Illustration of a bed topography survey in the studied river section

**Fig. 9:**
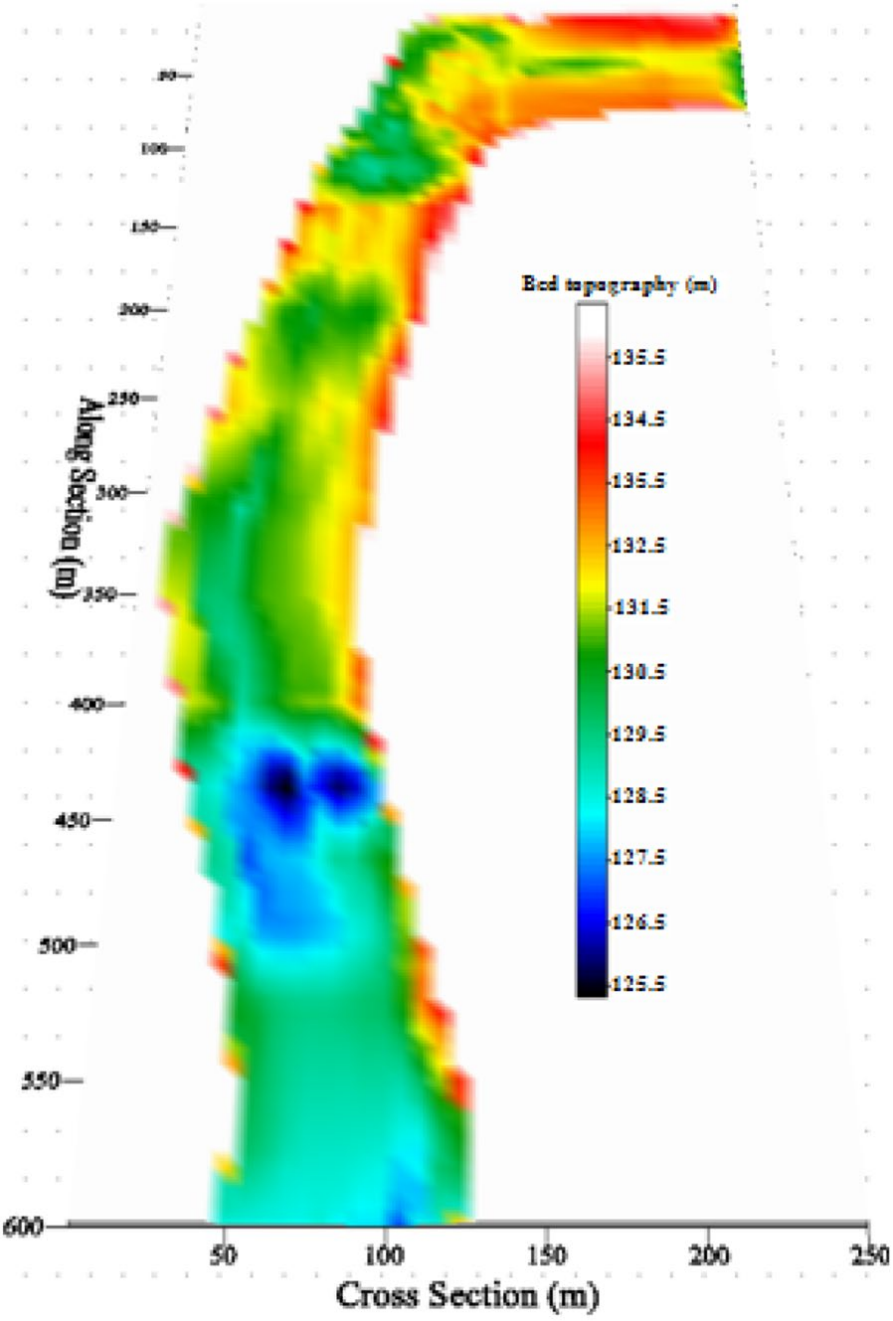
Bed topography and computational domain of the studied river section

Bed topography was collected from the Sokkia-C32 measuring device. All topographic and bathymetric data measured before and after the flood event (Fig. 8) are presented in the form of x-, y-, and z-points, corresponding to the longitude, latitude, and water depth of the computational domain (Fig. 9). They were then imported into an Excel file and interpolated into mesh points. They are based on the original data and interpolated mesh elevations. The surveys were then merged with archived data to form a single point data file in xyz form. Detailed information on the bed topography at an initial state is given in Fig. 9.

**Fig. 10:**
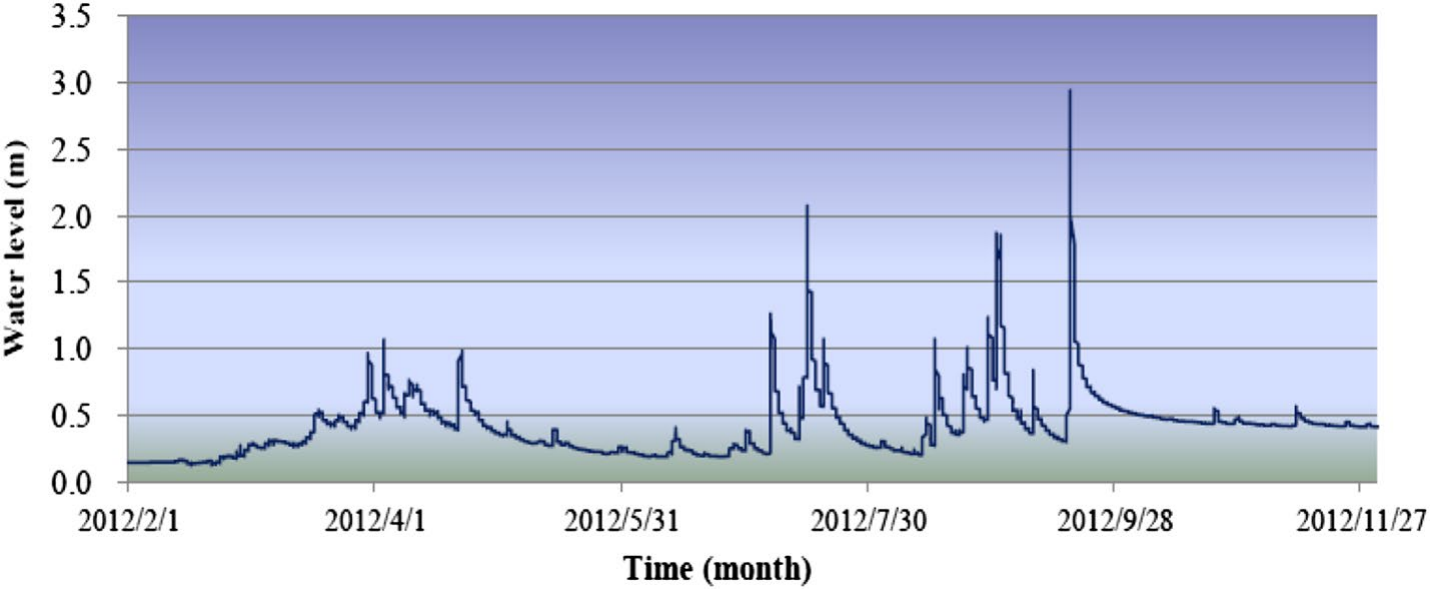
Illustration of the water level at the flow boundary

Simulation data were collected from January to November 2012 (Fig. 10). Water level stations were established at the inflow and outflow boundaries, and points inside of the study area were established to collect water level data using pressure sensor gauges. The location of each station was selected such that the study area was sufficiently covered to capture the water surface fluctuations with a high degree of accuracy.

**Fig. 11:**
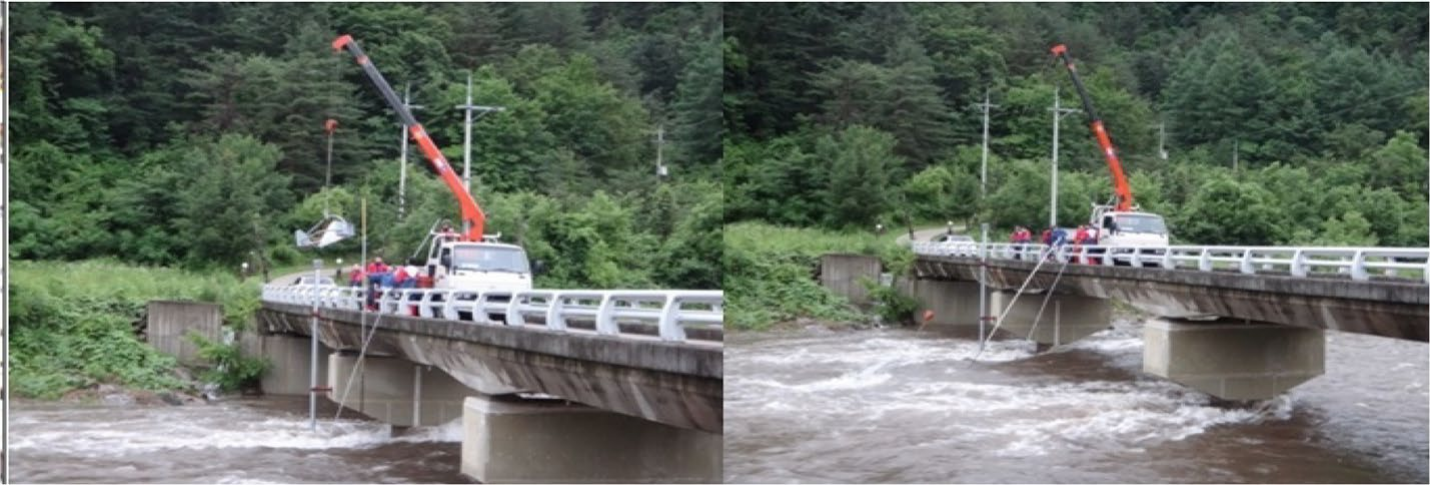
Illustration of a field survey to collect the bed load and suspended sediment

Field surveys were used to collect and analyze bed material sizes during a flood event (Fig. 11). The measured time series of sediment discharge at the inflow and outflow boundaries were established as boundary conditions.

According to Bravo-Espinosa et al. (2003), bed load transport conditions in alluvial channels are dependent on individual particle size fractions rather than on the complete spectrum of particle sizes represented by one characteristic particle size. Based on this viewpoint, to increase the accuracy of the sediment transport module in the numerical model, we divided the mean bed material size into several fractions based on data measured in the study area (Table 1).

**Table 1:** Distribution of bed material diameter fractions

Fraction	Material diameter (mm)	d_im_ (mm)	d_i_ (mm)
No.1	<0.01	0.005	0.005
No.2	0.125–0.5	0.31	0.3
No.3	0.5–2.0	1.25	1.25
No.4	2.0–8.0	5.0	5.0
No.5	8.0–16	12.0	10.0
No.6	16–32	24.0	25.0
No.7	32–64	48.0	50.0
No.8	64–128	95.0	100.0
No.9	128–265	196	150.0
No.10	>265	250	250.0

In Table 1, d_im_ is the material diameter and d_i_ is the mean material diameter.

**Fig. 12:**
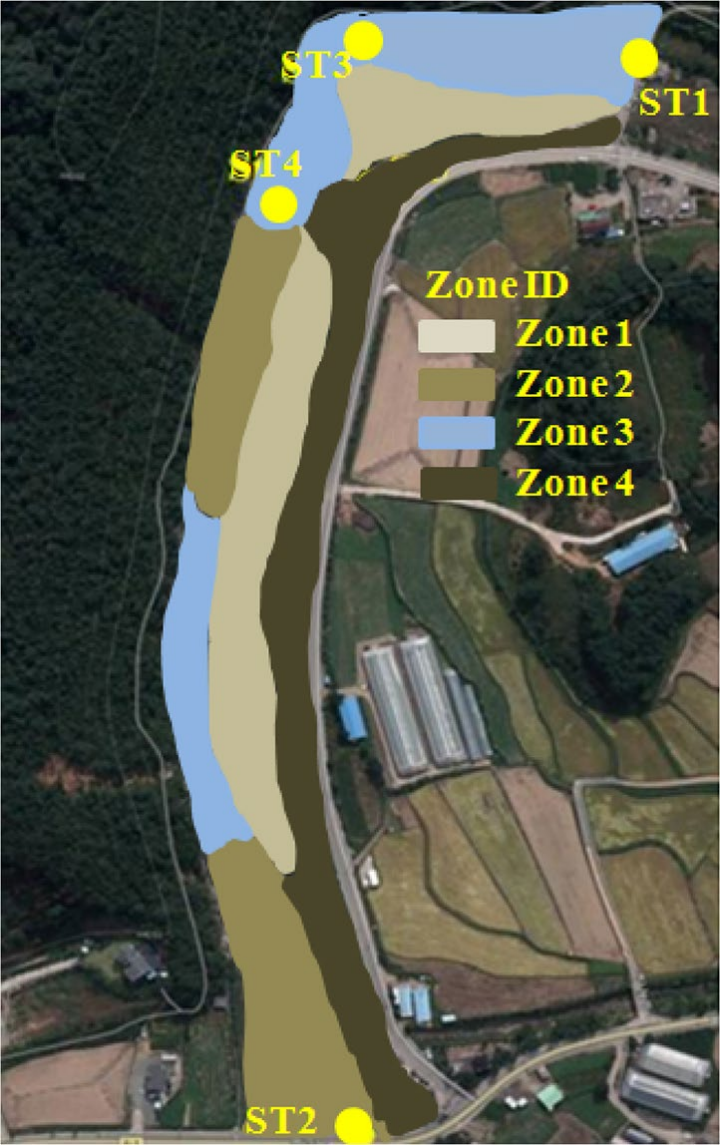
Illustration of the roughness zones used in the simulation

In this test case, the model was applied to simulate a flood event in the Asungjun River section, which has highly complex geometrical features. Therefore, the use of a single value for the Manning’s roughness coefficient is not appropriate because the channel bottom topography consists of widely different features. Thus, the computational domain in this study is divided into several different roughness zones, and Manning’s roughness coefficient is divided into several roughness zones. In Fig. 12, Zone 1 represents sand bars, Zones 2 represents gravel, Zone 3 represents boulders with heavy vegetation, and Zone 4 consists of boulders. Each zone was assigned a Manning’s coefficient that was determined through a calibration study by comparing the measured and predicted water levels. The calibrated Manning’s coefficients are listed in Table 2.

**Table 2:** Manning’s coefficients in the roughness zones shown in Fig. 13

Zone	Zone 1	Zone 2	Zone 3	Zone 4
Manning’s n	0.033	0.042	0.052	0.048

#### Wet/dry treatment

In shallow water regions of natural rivers where the water depth is small and the channel bed exhibits irregular geometry, the water edges change with time. In those cases, wet and dry treatments in numerical simulations are often used to determine the wet and dry cells. The water depth defined by the user will often depend on the scale of the simulation. In numerical models, the process of drying and wetting is represented by a flow domain that becomes dry when the water depth decreases and wet when the water depth increases.

Existing 2D models have taken a number of approaches to solve the problem associated with some areas being wet and others dry, or fluctuations between the two (Bates and Hervouet 1999; Begnudelli and Sanders 2006; DHI 2003). Several models turn cells on and off based on the minimum depth criteria (Delft 2002; King and Roig 1988; Leclerc et al. 1990). Other models change the fluid properties at very small depths so that a very thin layer of fluid is always present. Most approaches attempt to reformulate the flow equations over partially wet elements by introducing a scaling coefficient, representing the true volume of water at each element. This coefficient varies from zero to one as the cells tend from fully dry to fully wet (Bates and Hervouet 1999; Defina 2000).

In this study, a threshold value of the water depth (0.03 m) based on the river bed material size is used to establish drying and wetting. If the water depth in a cell is larger than this threshold value, this cell is considered wet, and if the water depth is lower than this threshold value, the cell is dry.

#### Results and discussion of the Asungjun River section case

**Fig. 13:**
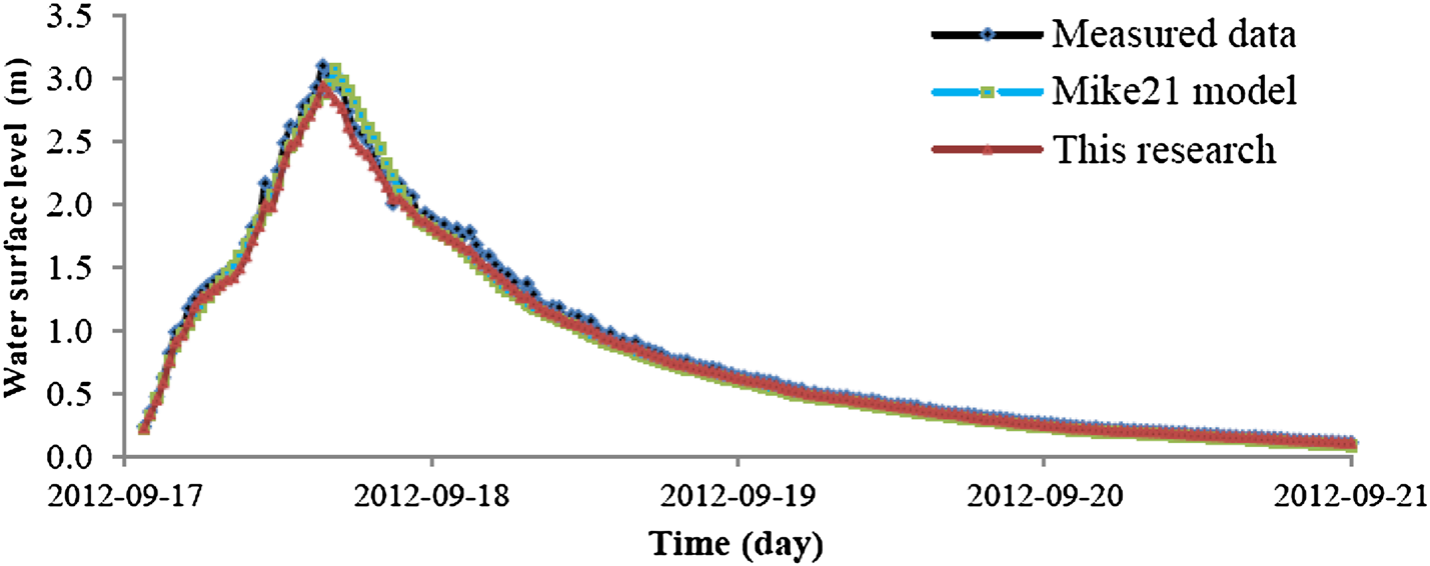
Comparison of the water level at the surveyed sites

The developed model was compared with the Mike1C model and calibrated using measured data. The simulation results of the developed model were slightly lower than those of the Mike21C model and survey data (Fig. 13). Additionally, the model slightly under predicted stages compared to stages predicted by Mike21C model and survey data. The simulation results of water level from the Mike21C model showed that the RMSD was 0.0039 and the ADM was 0.043, while the corresponding values of the developed model were 0.0032 and 0.030 (Table 3). Similarly, the Nash–Sutcliffe values between the measured water level and the Mike21C model and developed model were 0.89 and 0.97, respectively. This result suggests that the simulated model of the water level is very reliable.

**Table 3:** Comparison of results calculated between the numerical models

Model	Root mean square difference	Absolute difference mean
Developed model	0.0032	0.030
Mike21C model	0.0039	0.043

**Fig. 14:**
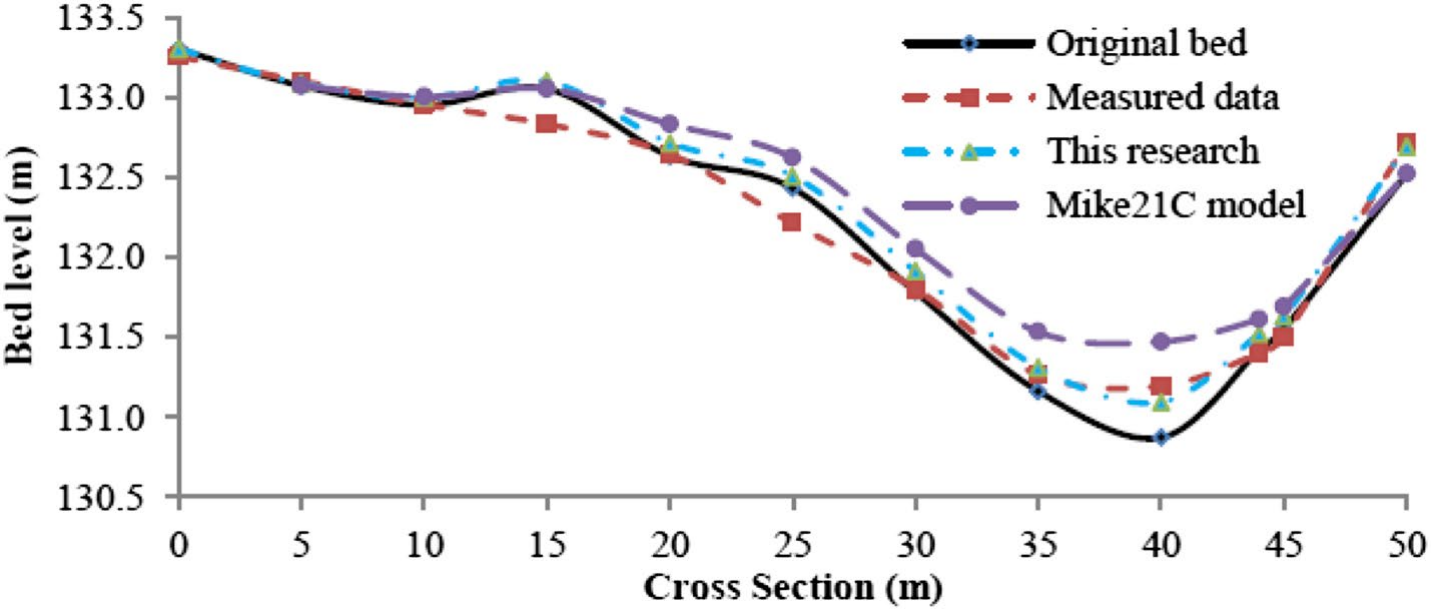
Final bed level after the flood event at cross section No.15 (present model and Mike21C)

**Fig. 15:**
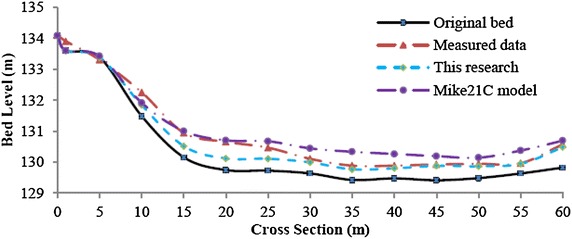
Final bed level after the flood event at cross section No.37 (present model and Mike21C)

Figure 14 shows a comparison of the developed model, Mike21C model, and measured bed elevation along several cross sections of the studied river section. The maximum aggradation at cross section No.15 was determined to be approximately 0.60 m compared with the original bed. This value was 0.22 m using the developed model and 0.32 m using the Mike21C model. Similarly, the maximum aggradation at cross section No.37 was calculated as 0.46 m compared to the original channel bed. At cross section No. 37, the value of the Mike21C model was 0.91 m and the value of the developed model was 0.34 m. The Nash–Sutcliffe values corresponding to the results of the Mike21C model and developed model were 0.76 and 0.97, respectively. This result suggests that the developed model is very reliable (Fig. 15). Generally, the simulation results of river morphology at different cross sections showed that the developed model was in good agreement with the observed data compared to the agreement of the Mike21C model.

**Fig. 16:**
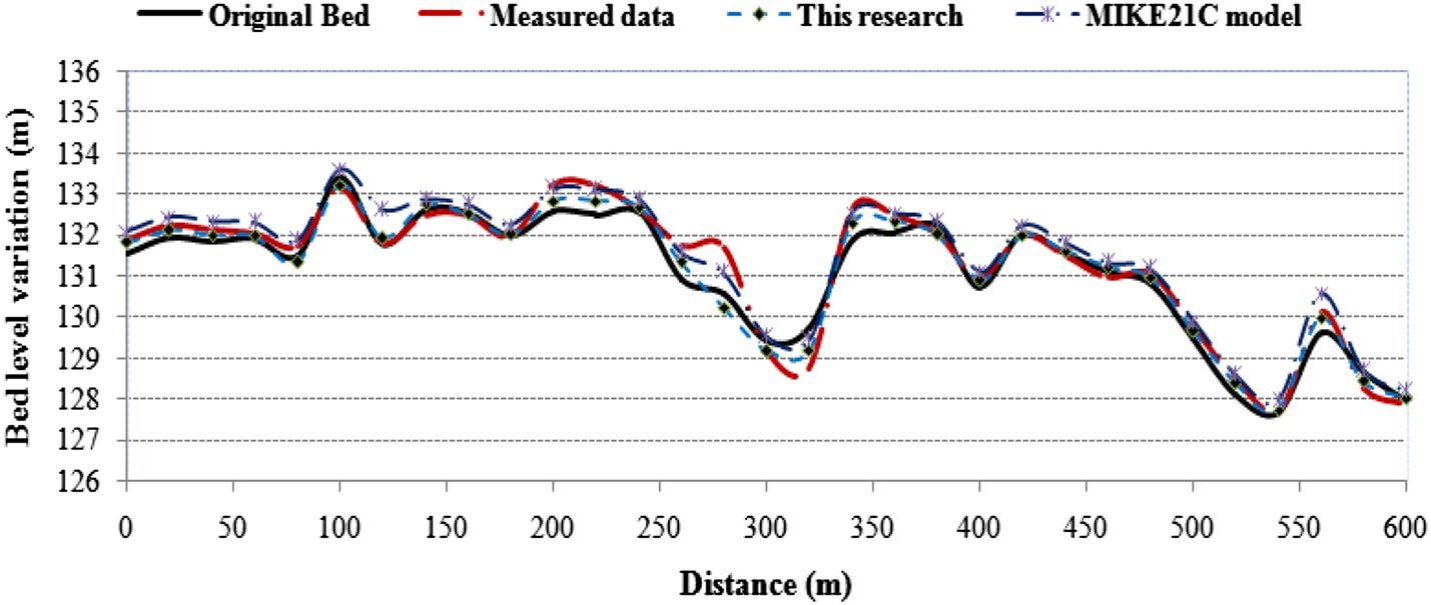
Final bed level after the flood event along the channel (present model and Mike21C)

Figure 16 shows the final bed level configuration after the flood event along the channel, and the differences between the simulation results of the developed model, the Mike21C model, and measured data are compared. The simulation results of the river morphology from Mike21C model exhibited an RMSD of 0.0028 and ADM of 0.035, while the corresponding values of the developed model were 0.0037 and 0.024. Similarly, the Nash–Sutcliffe criterion was used to validate the Mike21C model, developed model, and measured data. The Nash–Sutcliffe values corresponding to the Mike21C model and developed model were 0.81 and 0.96, respectively. These values confirm that the developed river morphology module is quite accurate.

## Conclusions

A depth-averaged 2D numerical model was developed for simulating river morphology in mountain rivers. FDM is used to solve the momentum equations and sediment continuity equations. The model system consists of flow and river morphology modules. Both the flow and river morphology modules are solved using an iteration method that constitutes a coupling procedure.

The bed material size distribution is separated using a fractional approach. This approach is more complex than the classical method, which only uses a value of particle size diameter (D_50_).

The simulation results of the river morphology of the flood event in the natural river using the developed model are more accurate than those produced by the Mike21C model. Generally, the simulation results were in good agreement with the measured data compared to the results of the Mike21C model.

The advantages of the developed model used for the simulation of the experimental channel and flood event are as follows:

- The robustness of the developed model under the various cases studied, such as lateral water and abrupt cross section variations, division of bed material into a number of size fractions, and division of Manning’s roughness coefficient into different values in study zones to fit the real bed topography conditions.
- The simple structure of the developed model allows users to easily control the calculation procedure, and it has a relatively fast computational speed.

The disadvantages of the developed model are as follows:

- The complicated procedure of constructing the numerical grid;
- The sensitivity of the coefficients to the river morphology;
- The need to calibrate multiple parameters when constructing the bed load formula.

More testing of the model may be necessary to improve its predictive ability. It is expected that the model will become a useful predictive tool for mountainous river studies.
