# Erratum to: Experimental analysis and numerical simulation of bed elevation change in mountain rivers

**DOI:** 10.1186/s40064-016-2890-1

**Published:** 2016-08-03

**Authors:** Truong An Dang, Sang Deog Park

**Affiliations:** 1 Sustainable Management of Natural Resources and Environment Research Group, Faculty of Environment and Labour Safety, Ton Duc Thang University, 19 Nguyen Huu Tho Str., Dist 7, Ho Chi Minh City, Vietnam; 2 Department of Civil Engineering, Gangneung-Wonju National University, Gangneung, Gangwon-Do 210‑702 South Korea

## Erratum to: SpringerPlus (2016) 5:107 DOI 10.1186/s40064-016-2714-3

Upon publication of the original article (Dang and Park 2016), it was noticed that the ‘Competing interests’ section was inaccurate. This has now been corrected in this erratum. Instead of:

“We received support from the Institute for Disaster Prevention, Gangneung-Wonju National University, South Korea.”

The text should read:

“This work was financially supported by a Grant (Code#’08 RTIP B-01) from Regional Technology Innovation Program funded by Ministry of Land, Transport and Maritime Affairs of Korean government.”

Furthermore, throughout the text, the word “Asungjun” should instead read “Eosungjun.” For example:

“The Asungjun River section of the mountainous Yangyang Namdae River (South Korea)” should read: “The Eosungjun River section of the mountainous Yangyang Namdae River (South Korea).”
